# Validation of Reference Genes for Real-Time Quantitative PCR (qPCR) Analysis of *Avibacterium paragallinarum*

**DOI:** 10.1371/journal.pone.0167736

**Published:** 2016-12-12

**Authors:** Shuxiang Wen, Xiaoling Chen, Fuzhou Xu, Huiling Sun

**Affiliations:** Beijing Key Laboratory for Prevention and Control of Infectious Diseases in Livestock and Poultry, Institute of Animal Husbandry and Veterinary Medicine, Beijing Academy of Agriculture and Forestry Sciences, Beijing, China; Texas Technical University Health Sciences Center, UNITED STATES

## Abstract

Real-time quantitative reverse transcription PCR (qRT-PCR) offers a robust method for measurement of gene expression levels. Selection of reliable reference gene(s) for gene expression study is conducive to reduce variations derived from different amounts of RNA and cDNA, the efficiency of the reverse transcriptase or polymerase enzymes. Until now reference genes identified for other members of the family *Pasteurellaceae* have not been validated for *Avibacterium paragallinarum*. The aim of this study was to validate nine reference genes of serovars A, B, and C strains of *A*. *paragallinarum* in different growth phase by qRT-PCR. Three of the most widely used statistical algorithms, geNorm, NormFinder and ΔCT method were used to evaluate the expression stability of reference genes. Data analyzed by overall rankings showed that in exponential and stationary phase of serovar A, the most stable reference genes were *gyrA* and *atpD* respectively; in exponential and stationary phase of serovar B, the most stable reference genes were *atpD* and *recN* respectively; in exponential and stationary phase of serovar C, the most stable reference genes were *rpoB* and *recN* respectively. This study provides recommendations for stable endogenous control genes for use in further studies involving measurement of gene expression levels.

## Introduction

Infectious coryza is an acute upper respiratory tract disease of chickens. This disease is of worldwide economic significance and affects both broiler and layer flocks, manifested primarily as a drop in egg production (10±40%) in layer flocks and retardation of growth due to decreased feed and water consumption in breeder and broiler flocks. The most common clinical signs are nasal discharge, conjunctivitis, facial oedema, lacrimation, anorexia, and diarrhea [1].

The causative agent of infectious coryza is Avibacterium paragallinarum (*A*. *paragallinarum*) [2], and *A*. *paragallinarum* is classified into three serovars: A, B, and C according to the Page schemes [3]. It is widely accepted that the three Page serovars represent distinct “immunovars,” since inactivated vaccines based on any one Page serovar provide no protection against the other two Page serovars [4]. However, little is known about the differences among these three serovars, either the genes defining each serovar or the expression of these genes.

Real-time quantitative reverse transcription PCR (qRT-PCR) is a robust and sensitive method for measurement of gene expression and characteraization of gene regulation. In most qPCR studies, internal reference genes are used to eliminate sample-to-sample variations that may arise due to test variation including differences in cell numbers and efficiency of RNA isolation and reverse transcription [5]. Since “housekeeping” metabolism of prokaryotes is highly variable depending on experimental procedures [6], selection of reference genes is crucial for the accuracy of a qRT-PCR test. Once the reference genes are selected, any changes in target gene expression can be expressed in relation to those of the reference genes. A single gene is often selected as the reference gene but Vandesompele et al [7] suggested that multiple carefully selected housekeeping genes were recommendable and more suitable for accurate normalization.

Development of an effective qPCR for defining gene expression in serovars A, B, and C of *A*. *paragallinarum* is urgently needed. However, information concerning reference genes as candidates for a qPCR against *A*. *paragallinarum* is very limited, primarily due to a lack of understanding the genome organization of serovars A, B, and C of *A*. *paragallinarum*. In this study, nine candidate reference genes encoding 16S ribosomal subunit (*16S rRNA*), the DNA gyrase subunit A (*gyrA*), theβ-subunit of RNA polymerase (*rpoB*), the glucose-6-phosphate isomerase (*pgi*), the DNA repair protein (*recN*), the translation initiation factor 2 (*infB*), the DNA gyrase subunit B (*gyrB*), the β-subunitof the ATP synthase (*atpD*) and the Mn-dependent superoxide dismutase (*sodA*), respectively, were chosen for validating the reference genes for qPCR of *A*. *paragallinarum*. The majority of these genes were recognized as housekeeping genes in the family of *Pasteurellaceae* and used in phylogenetic analysis [8–13]. Moreover, five of the nine genes (including 16S rRNA, gyrA, rpoB, *atpD* and gyrB) were also used for qRT-PCR normalization in *Actinobacillus suis* and *Haemophilus ducreyi* [14,15].

To date, no study has systematically investigated reference genes for A. paragallinarum. In order to verify the stable expression genes and determine whether they are suitable for normalization of qPCR data for *A*. *paragallinarum*, we performed molecular biological analysis of their expression stability. The objective of this work was to validate internal reference genes for a qRT-PCR of serovar A, B, and C strains of *A*. *paragallinarum*. The expression of nine reference genes was examined during different growth phase. The results of this study will be helpful for gene expression normalization of qPCR in serovars A, B, and C of *A*. *paragallinarum*.

## Materials and Methods

### Bacterial cultures and sample processing

The three reference strains 221, 0222, and Modesto of the *A*. *paragallinarum* serovar A, B, and C respectively were kindly provided by Dr. Pat Blackall, University of Queensland, Australia. Tryptic Soya Broth (TSB) and Tryptic Soya Broth Agar (TSA), supplemented with 10% (v/v) chicken serum and 0.0025% (w/v) reduced Nicotinamied adenine dinucleotide (NAD) were used for propagation and maintenance of these three strains. The cultures were grown in a shaking incubator at 37°C. Broth Cultures of serovar A, B and C were monitored by OD600 measurement every 40 min, and samples were harvested from the exponential and stationary phase by centrifugation, followed immediately resuspended in RNAlater (Ambion, Carlsbad, CA). Samples were then stored at 4°C until the test.

### Primer design and validation

Primers were designed from GenBank sequences with the aid of primer analysis software Primer3plus (http://www.primer3plus.com/cgi-bin/dev/primer3plus.cgi; Version 2.4.0) [16,17]. Then confirmed through DNAMAN and NCBI/Primer-BLAST. Characteristics of the primers are listed in Table 1. The length of the amplicons were kept between 100–250 bp as much as possible for optimal amplification efficiency. The effectiveness of the primers was confirmed by conventional PCR and product size observed by electrophoresis on 1.5% agarose gels. All nine primers produced single amplification products as expected (data not show).

**Table 1:** Information of the primers and corresponding candidate reference genes.

Gene	Accession No.	Correlation (r^2^)	Forward (F) and Reverse (R) Primers	Efficiency %	Amplicon length
**gyrB**	**NZ_LAEN01000056.1**	**0.989**	**F:CAACTTCATCGCCCATTAGG;R:GGGAGAAATGAACCCAGAAC**	**88.2**	**191**
**recN**	**JN592546.1**	**0.981**	**F:AGCTTGCTCTACCGCACAAT;R:CTGGCTTCTTGCACCTGAAT**	**102.1**	**113**
**rpoB**	**NZ_AFFP02000004.1**	**0.994**	**F:GCTTAATGCCGCTTCACCTA;R:AGCGTGTGGTGCAAGAAGAT**	**99.1**	**131**
**infB**	**EU350938.1**	**0.999**	**F:GCCAGTTGCTACCATTTTGG;R:AGCCTAGCACTTCCACAGGA**	**97.3**	**155**
**pgi**	**JN592536.1**	**0.999**	**F:GGAAAGGCTACACAGGCAAA;R:AACACAAGGGTGGTTTCTGG**	**96.7**	**196**
**sodA**	**DQ005620.1**	**0.999**	**F:TTAGCAGAAGTGCCAGCAGA;R:GCTTCCACAGAACCGAAATC**	**92.7**	**155**
**atpD**	**AF326327.1**	**0.993**	**F:TCCCACAAGATGCAGTACCA;R:CCCACTGGAACAGAAATTGG**	**112.0**	**181**
**gyrA**	**NZ_AFFP02000003.1**	**0.993**	**F:AGTGAGCGTAACGGCAAAGT;R:ATGTCCGATTCTTCGTCGTC**	**98.7**	**218**
**16S rRNA**	**KF280244.1**	**0.997**	**F:AGGCCTTCGGGTTGTAAAGT;R:CGGGGATTTCACATCTCACT**	**99.2**	**201**

### RNA extraction and cDNA synthesis

Total RNA was extracted from 0.5 ml aliquot of bacterial samples collected at different growth phase using Trizol RT extraction system (Invitrogen, carlsbad, CA) following the manufacturer’s instruction. The extracted RNA was re-suspended in DEPC-treated water (Life Technologies) and the concentration and purity of RNA were determined by NanoDrop1000® spectrophotometer (NanoDrop Technologies Inc, Wilmington, DE, USA). RNA samples with 260nm/280nm ratio between 1.9 and 2.1 were prepared in equimolar aliquots for further tests. cDNA was synthesized from 200 ng of each RNA sample [18] using a Reverse Transcription Kit (Tiangen, China). Prior to cDNA synthesis, genomic DNA (gDNA) in the RNA samples were removed by incubation with a gDNA buffer at 42°C for 3 min as described in RNA reverse transcription kit (Tiangen, China). Reverse transcription reactions were performed in a MasterCycler ® Gradient Thermal Cycler under the following conditions: 42°C for 15 min, 95°C for 3 min. The cDNA samples were placed immediately on ice at the end of the reactions and then stored at -20°C for later use.

### Real-time quantitative PCR

Real-time quantitative PCR (qRT-PCR) was performed with Bio-rad IQ5 (Bio-Rad, Hercules, CA) using 2X SYBR Green iTaq mixture (Tiangen, China) in a total reaction volume of 12.5 ul. The reaction mixture consisted of: 6.25 ul of 2X SYBR Green iTaqmixture, 0.25 ul forward/reverse primer mix with an initial concentration of 10 uM, 1 ul of cDNA (1:2 dilution) and DEPC-treated water added to12.5 ul. Each sample was tested in triplicate. The cycling condition was as follows: 3 min denaturation at 95°C, followed by 40 cycles at 94°C for 40 s, 56/58°C for 40 s and 72°C for 40 s.

### Data and statistical analysis

Cycle threshold (CT) values, also known as Cq recommended by Bustin [19], were recorded for all qPCR reactions. Two of the most widely used statistical algorithms, geNorm v3.5 and NormFinder [20,21] were used to evaluate the expression stability of reference genes. The comparative ΔCT method [22] was used to rank candidate reference genes, with the lowest standard deviation considered to signify the highest stability. The geNorm algorithm determines the most stable combination of reference targets based on the geometric mean of the most stable control genes to generate a stability value (M). The goal of the alanysis was to choose two or more reference genes to obtain more reliable quantitative results according to the pairwise variance analysis of normalization factor (V_n/n+1_). NormFinder Excel applet, as a similar calculation method as geNorm and also based on relative expression levels, was used to assess reference gene stability based on both intra- and inter- group variations. NormFinder was used to identify genes with the lowest standard deviation (SV) as an indication for highest stability. The comparative ΔCT method was subsequently used to further evaluate gene expression stability. This method compares the relative expression of pairs of genes within each treatment and selects the most stable reference gene by assessing expression stability based on standard deviation derived from CT values. If the ΔCT values between pairs of genes remain constant for all samples tested, it means these two genes are either stably expressed or co-regulated. However, if the ΔCT values vary logarithmically, as reflected by higher standard deviations, it indicates that one of these two reference genes is variably transcribed. In such event, ΔCT analysis was performed to compare each gene with all other genes and standard deviation of each gene was obtained. The reference gene with the lowest standard deviation was then selected as the most stable reference gene. Overall ranking of reference genes was confirmed by using the geometric mean of the rankings generated from the individual algorithms [23].

### Analysis of gene expression using different reference genes for normalization

In order to evaluate the impact of using different reference gene for normalization on the expression levels measured by qRT-PCR, the two best-ranked, the two middle-ranked reference genes and a least-ranked reference genes were used to calculate the expression levels of hypothetical gene of interest along with the growth time for different serovars of *A*. *paragallinarum*. Expression levels of the hypothetical gene of interest were generated with normalization using the most stable reference genes, the stable reference genes and the least stable reference gene. The effect of reference genes with different ranks of stability was assessed by variation tendency of expression level for the hypothetical gene of interest along with the growth time for strains of *A*. *paragallinarum*.

## Results

### Primer amplification efficiency

The efficiency, linear dynamic range and specificity of nine pairs of primers were evaluated in qPCR with a series of five-fold dilution starting at 1:5 for a total of 5 dilutions. The efficiencies (E) of all primer pairs ranged from 88.2 to 112%, and correlation coefficients (R^2^) were all higher than 0.98 (Table 1), both being considered acceptable [6,24]. Primer specificity was verified by the presence of a single-peak in the melting curve analysis in qRT-PCR (data not shown).

### Expression profiles of candidate reference genes

**Fig 1:**
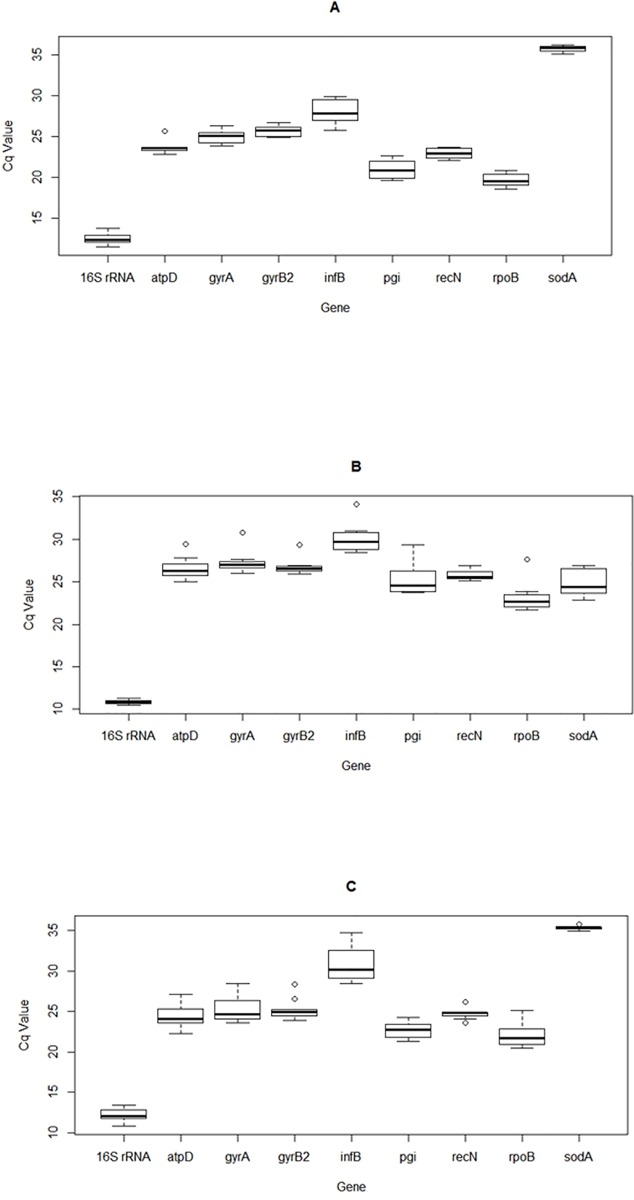
**Range of Cq values of the nine candidate reference genes across all samples of *A*. *paragallinarum* serovars A, B, and C.** Boxes and whiskers represent interquartile ranges and confidence intervals. Bars inside boxes indicate median values. Hollow circles show outliers (5th/95th percentile) respectively.

In this study, certain variations in the expression levels of the nine candidate reference genes were observed in serovars A, serovars B and serovars C as shown in Fig 1A, Fig 1B and Fig 1C, respectively. The higher the level of gene expressed, the smaller the Cq value was. The Cq values of candidate genes ranged widely (10.48–36.24) in all tested samples. It was notable that the gene encoding superoxide dismutase (*sodA* gene) had the lowest expression levels in serovar A and C, reaching a cycle threshold after 35 amplification cycles and there was a significant difference in Cq value among serovars A, B, and C, while the mean Cq value of the *sodA* gene in serovar B was 24.27. The gene encoding 16S rRNA was highly expressed in all three serovars compared to the other genes, reaching a cycle threshold after only 10.48 amplification cycles.

### GeNorm analysis

The expression stabilities of the nine candidate reference genes were analyzed using geNorm algorithms. High gene expression variability results in high M values and indicates low expression stability. For overall comparison, samples from three serovars at each growth phase (exponential and stationary) were calculated. In cultures of serovar A, *gyrA* (M = 0.468 and 0.428) was found to be most stably expressed gene while *sodA* (M = 1.781 and 1.257) was the least stably expressed both in exponential and stationary growth phase (Table 2). In cultures of serovar B, the most stable genes were found to be *atpD* (M = 0.438) and *recN* (M = 0.355) in exponential and stationary growth phase respectively, and the least stable gene was *sodA* (M = 1.046 and 0.686) (Table 3). In cultures of serovar C, *rpoB* (M = 0.347) and *recN* (M = 0.436) were found to be the most stably expressed genes in the exponential and stationary growth phase respectively while *sodA* (M = 1.154 and 1.026) was the least stably expressed both in the exponential and the stationary growth phase (Table 4). When combining exponential and the stationary phase, the most stable reference genes were 16S rRNA, *recN* and *gyrA*.

**Table 2:** Gene expression stability rankings for different growth phase in *A*. *paragallinarum* serovar A analyzed by geNorm.

Exponential		Stationary		Combined	
**gyrA**	**0.468**	**gyrA**	**0.428**	**16S rRNA**	**0.572**
**recN**	**0.480**	**atpD**	**0.436**	**gyrA**	**0.583**
**16S rRNA**	**0.513**	**rpoB**	**0.450**	**recN**	**0.590**
**rpoB**	**0.542**	**recN**	**0.470**	**rpoB**	**0.596**
**pgi**	**0.562**	**gyrB**	**0.484**	**pgi**	**0.604**
**atpD**	**0.606**	**infB**	**0.613**	**gyrB**	**0.666**
**gyrB**	**0.609**	**16S rRNA**	**0.625**	**atpD**	**0.673**
**infB**	**0.750**	**pgi**	**0.861**	**infB**	**1.009**
**sodA**	**1.781**	**sodA**	**1.257**	**sodA**	**2.040**

**Table 3:** Gene expression stability rankings for different growth phase in *A*. *paragallinarum* serovar B analyzed by geNorm.

Exponential		Stationary		Combined	
**atpD**	**0.438**	**recN**	**0.355**	**recN**	**0.516**
**gyrA**	**0.447**	**16S rRNA**	**0.371**	**16S rRNA**	**0.554**
**recN**	**0.458**	**pgi**	**0.383**	**gyrA**	**0.556**
**16S rRNA**	**0.523**	**gyrA**	**0.384**	**atpD**	**0.593**
**pgi**	**0.530**	**rpoB**	**0.438**	**gyrB**	**0.644**
**gyrB**	**0.571**	**gyrB**	**0.476**	**infB**	**0.684**
**infB**	**0.602**	**atpD**	**0.488**	**pgi**	**0.711**
**rpoB**	**0.673**	**infB**	**0.600**	**rpoB**	**0.801**
**sodA**	**1.046**	**sodA**	**0.686**	**sodA**	**1.539**

**Table 4:** Gene expression stability rankings for different growth phase in *A*. *paragallinarum* serovar C analyzed by geNorm.

Exponential		Stationary		Combined	
**rpoB**	**0.347**	**recN**	**0.436**	**gyrA**	**0.502**
**recN**	**0.365**	**gyrB**	**0.440**	**recN**	**0.502**
**gyrA**	**0.378**	**rpoB**	**0.506**	**gyrB**	**0.504**
**gyrB**	**0.395**	**gyrA**	**0.515**	**rpoB**	**0.536**
**16S rRNA**	**0.429**	**pgi**	**0.519**	**16S rRNA**	**0.580**
**pgi**	**0.471**	**atpD**	**0.575**	**atpD**	**0.593**
**infB**	**0.542**	**16S rRNA**	**0.584**	**pgi**	**0.617**
**atpD**	**0.561**	**infB**	**0.825**	**infB**	**0.747**
**sodA**	**1.154**	**sodA**	**1.026**	**sodA**	**0.877**

In addition to the ranking of the candidate reference genes, geNorm also recommends using optimal number of required reference genes and provides calculations of the impact of adding additional reference genes on normalization (V_n_/_n+1_). If a pairwise value (V_n_/_n+1_) is no more than 0.15, then there is no need to choose n+1 reference genes. In our study, the best combination of reference genes assessed by geNorm analysis was *gyrA* and *recN*, *atpD* and *gyrA*, *recN* and *rpoB* in exponential growth phase for serovars A, B, and C respectively (with V_2/3_ = 0.053, 0.12, and 0.053, respectively). In stationary growth phase of serovars A, B, and C, however, the stability ranking-order seemed to vary with serovars. The optimal numbers and best combinations of reference genes in this phase were *gyrA* and *rpoB*, *gyrA* and *pgi*, *rpoB* and *atpD* for serovars A, B, and C respectively (with V2/3 = 0.052, 0.101, and 0.085, respectively). When combining the exponential and stationary growth phase, the optimal numbers and best combinations of reference genes were *recN* and 16S rRNA, *recN* and 16S rRNA, *recN*, *rpoB* and *gyrA* for serovars A, B, and C respecitively (with V2/3 = 0.086, V2/3 = 0.110, and V3/4 = 0.096, respectively).

### NormFinder analysis

Stabilities of the expression of the nine reference genes were evaluated using NormFinder analysis. A lower stability value indicates a more stably expressed reference gene. NormFinder also suggests the best combination of two reference genes for quantitative real-time PCR normalization. For serovar A cultures, NormFinder identified *gyrA*, *atpD*, and *gyrA* as the most stable reference genes in exponential, stationary, and combined both growth phase, respectively (Table 5). For serovar B cultures, the most stable genes were found to be *atpD*, *recN*, and *gyrA* in the three growth phase respectively (Table 6). For serovar C cultures, the most stable genes were found to be *gyrA*, *gyrB*, and *gyrB* in the three growth phase respectively (Table 7).

**Table 5:** Gene expression stability rankings for different growth phase in *A*. *paragallinarum* serovar A analyzed by NormFinder.

Exponential		Stationary		Combined	
**gyrA**	**0.028**	**atpD**	**0.036**	**gyrA**	**0.047**
**recN**	**0.028**	**gyrB**	**0.039**	**16S rRNA**	**0.062**
**16S rRNA**	**0.056**	**gyrA**	**0.042**	**recN**	**0.062**
**gyrB**	**0.063**	**rpoB**	**0.042**	**pgi**	**0.084**
**pgi**	**0.170**	**recN**	**0.120**	**gyrB**	**0.091**
**rpoB**	**0.206**	**infB**	**0.275**	**rpoB**	**0.157**
**atpD**	**0.333**	**16S rRNA**	**0.343**	**atpD**	**0.324**
**infB**	**0.483**	**pgi**	**0.568**	**infB**	**0.663**
**sodA**	**1.229**	**sodA**	**0.853**	**sodA**	**1.408**

**Table 6:** Gene expression stability rankings for different growth phase in *A*. *paragallinarum* serovar B analyzed by NormFinder.

exponential		stationary		Combined	
**atpD**	**0.030**	**recN**	**0.016**	**gyrA**	**0.042**
**gyrA**	**0.050**	**16S rRNA**	**0.081**	**recN**	**0.043**
**recN**	**0.134**	**pgi**	**0.136**	**16S rRNA**	**0.124**
**pgi**	**0.204**	**gyrA**	**0.137**	**atpD**	**0.198**
**16S rRNA**	**0.263**	**rpoB**	**0.227**	**pgi**	**0.252**
**infB**	**0.283**	**gyrB**	**0.253**	**gyrB**	**0.274**
**gyrB**	**0.285**	**atpD**	**0.273**	**infB**	**0.331**
**rpoB**	**0.420**	**infB**	**0.391**	**rpoB**	**0.496**
**sodA**	**0.705**	**sodA**	**0.458**	**sodA**	**1.051**

**Table 7:** Gene expression stability rankings for different growth phase in *A*. *paragallinarum* serovar C analyzed by NormFinder.

exponential		stationary		Combined	
**gyrA**	**0.007**	**gyrB**	**0.056**	**gyrB**	**0.152**
**recN**	**0.029**	**recN**	**0.056**	**gyrA**	**0.154**
**rpoB**	**0.029**	**gyrA**	**0.142**	**recN**	**0.161**
**gyrB**	**0.055**	**pgi**	**0.172**	**rpoB**	**0.229**
**16S rRNA**	**0.134**	**rpoB**	**0.219**	**16S rRNA**	**0.259**
**pgi**	**0.227**	**16S rRNA**	**0.273**	**atpD**	**0.294**
**infB**	**0.304**	**atpD**	**0.311**	**pgi**	**0.323**
**atpD**	**0.338**	**infB**	**0.553**	**infB**	**0.460**
**sodA**	**0.788**	**sodA**	**0.699**	**sodA**	**0.553**

### ΔCT analysis

The comparative ΔCT method was used to assess the best reference gene by comparing standard deviation of a particular gene with all other genes. The results of the comparative ΔCT method analysis were similar to those from geNorm and Normfinder. However, both major and minor differences still exist in the tested samples from the other two analysis methods. A summary of the full results can be seen in Table 8.

**Table 8:** Gene expression stability assessed by the comparative ΔCT method.

Serovar A						Serovar B						Serovar C					
Exponential		Stationary		Combined		Exponential		Stationary		Combined		Exponential		Stationary		Combined	
gene	Mean s.d	gene	Mean s.d	gene	Mean s.d	gene	Mean s.d	gene	Mean s.d	gene	Mean s.d	gene	Mean s.d	gene	Mean s.d	gene	Mean s.d
**pgi**	**0.32**	**atpD**	**0.32**	**gyrB**	**1.70**	**16S rRNA**	**0.41**	**recN**	**0.36**	**16S rRNA**	**0.24**	**rpoB**	**0.24**	**recN**	**0.35**	**gyrB**	**0.44**
**rpoB**	**0.34**	**gyrA**	**0.33**	**recN**	**2.36**	**atpD**	**0.44**	**pgi**	**0.38**	**recN**	**0.31**	**recN**	**0.26**	**gyrB**	**0.38**	**recN**	**0.56**
**16S rRNA**	**0.36**	**recN**	**0.35**	**gyrA**	**2.45**	**gyrA**	**0.45**	**gyrA**	**0.38**	**gyrB**	**0.35**	**gyrA**	**0.28**	**rpoB**	**0.40**	**rpoB**	**0.62**
**gyrA**	**0.39**	**rpoB**	**0.35**	**rpoB**	**2.50**	**recN**	**0.46**	**16S rRNA**	**0.44**	**gyrA**	**0.41**	**gyrB**	**0.32**	**gyrA**	**0.46**	**gyrA**	**0.63**
**gyrB**	**0.40**	**gyrB**	**0.41**	**16S rRNA**	**2.50**	**rpoB**	**0.50**	**gyrB**	**0.48**	**rpoB**	**0.56**	**16S rRNA**	**0.34**	**atpD**	**0.47**	**16S rRNA**	**0.63**
**atpD**	**0.43**	**16S rRNA**	**0.54**	**atpD**	**2.74**	**pgi**	**0.53**	**atpD**	**0.49**	**atpD**	**0.56**	**pgi**	**0.36**	**pgi**	**0.49**	**pgi**	**0.69**
**recN**	**0.45**	**infB**	**0.56**	**pgi**	**2.81**	**gyrB**	**0.57**	**infB**	**0.60**	**infB**	**0.65**	**infB**	**0.43**	**16S rRNA**	**0.59**	**atpD**	**0.77**
**infB**	**0.73**	**pgi**	**0.73**	**infB**	**2.91**	**infB**	**0.60**	**rpoB**	**0.61**	**pgi**	**0.70**	**atpD**	**0.44**	**infB**	**0.71**	**infB**	**0.92**
						**sodA**	**1.05**	**sodA**	**0.69**	**sodA**	**1.63**						

### Ranking of candidate reference genes

In order to mitigate potential biases introduced by any single calculation method, we developed a composite ranking based on geometric mean of the results from all three algorithms described above. The lower the geometric average is, the more stable of the candidate reference gene expresses. Here we briefly described our findings for different growth phase and different serovars. In serovar A cultures, the overall rankings are *gyrA*>*pgi*>*16S rRNA*>*recN*>*rpoB*>*gyrB*>*atpD*>*infB* in exponential phase, followed by *atpD*>*gyrA*>*rpoB*>*gyrB*>*recN*>*infB*>*16S rRNA*>*pgi* in stationary phase, then *gyrA*>*16S rRNA*>*recN*>*gyrB*>*rpoB*>*pgi*>*infB* in combined both growth phase (Table 9). In serovar B cultures, the overall rankings are *atpD*>*gyrA*>*16S rRNA*>*recN*>*pgi*>*gyrB*>*rpoB*>i*nfB*>*sodA* in exponential phase, followed by *recN*>*16S rRNA*>*pgi*>*gyrA*>*gyrB*>*rpoB*>*atpD*>*infB*>s*odA* in stationary phase, then *recN*>*16S rRNA*>*gyrA*>*gyrB*>*atpD*>*pgi*>i*nfB*>*rpoB*>*sodA* in combined both growth phase (Table 10). In serovar C cultures, the overall rankings are *rpoB*>*recN*>*gyrA*>*gyrB*>*16S rRNA*>*pgi*>*infB*>*atpD* in exponential phase, followed by *recN*>*gyrB*>*rpoB*>*gyrA*>*pgi*>*atpD*>*16S rRNA*>*infB* in stationary phase, then *gyrB*> *gyrA*> *recN*> *rpoB*>*16S rRNA*> *atpD*> *pgi*>*infB* in combined both growth phase (Table 11).

**Table 9:** Candidate reference genes ranked by different methods in serovar A.

Exponential					Stationary					Combined				
geN	Norm	ΔCT	Ranking	Mean	geN	Norm	ΔCT	Ranking	Mean	geN	Norm	ΔCT	Ranking	Mean
**gyrA**	**gyrA**	**pgi**	**gyrA**	**1.59**	**gyrA**	**atpD**	**atpD**	**atpD**	**1.26**	**16S rRNA**	**gyrA**	**gyrB**	**gyrA**	**1.82**
**recN**	**recN**	**rpoB**	**pgi**	**2.92**	**atpD**	**gyrB**	**gyrA**	**gyrA**	**1.82**	**gyrA**	**16S rRNA**	**recN**	**16S rRNA**	**2.15**
**16S rRNA**	**16S rRNA**	**16S rRNA**	**16S rRNA**	**3.00**	**rpoB**	**gyrA**	**rpoB**	**rpoB**	**3.30**	**recN**	**recN**	**gyrA**	**recN**	**2.62**
**rpoB**	**gyrB**	**gyrA**	**recN**	**3.04**	**recN**	**rpoB**	**recN**	**gyrB**	**3.42**	**rpoB**	**pgi**	**rpoB**	**gyrB**	**3.11**
**pgi**	**pgi**	**gyrB**	**rpoB**	**3.63**	**gyrB**	**recN**	**gyrB**	**recN**	**3.91**	**pgi**	**gyrB**	**16S rRNA**	**rpoB**	**4.58**
**atpD**	**rpoB**	**atpD**	**gyrB**	**5.19**	**infB**	**infB**	**16S rRNA**	**infB**	**6.00**	**gyrB**	**rpoB**	**atpD**	**pgi**	**5.19**
**gyrB**	**atpD**	**recN**	**atpD**	**6.32**	**16S rRNA**	**16S rRNA**	**infB**	**16S rRNA**	**6.26**	**atpD**	**atpD**	**pgi**	**atpD**	**6.65**
**infB**	**infB**	**infB**	**infB**	**8.00**	**pgi**	**pgi**	**pgi**	**pgi**	**7.65**	**infB**	**infB**	**infB**	**infB**	**8.00**

**Table 10:** Candidate reference genes ranked by different methods in serovar B.

Exponential					Stationary					Combined				
geN	Norm	ΔCT	Ranking	Mean	geN	Norm	ΔCT	Ranking	Mean	geN	Norm	ΔCT	Ranking	Mean
**atpD**	**atpD**	**16S rRNA**	**atpD**	**1.26**	**recN**	**recN**	**recN**	**recN**	**1.00**	**recN**	**gyrA**	**16S rRNA**	**recN**	**1.59**
**gyrA**	**gyrA**	**atpD**	**gyrA**	**2.29**	**16S rRNA**	**16S rRNA**	**pgi**	**16S rRNA**	**2.29**	**16S rRNA**	**recN**	**recN**	**16S rRNA**	**1.82**
**recN**	**recN**	**gyrA**	**16S rRNA**	**2.71**	**pgi**	**pgi**	**gyrA**	**pgi**	**2.62**	**gyrA**	**16S rRNA**	**gyrB**	**gyrA**	**2.29**
**16S rRNA**	**pgi**	**recN**	**recN**	**3.30**	**gyrA**	**gyrA**	**16S rRNA**	**gyrA**	**3.17**	**atpD**	**atpD**	**gyrA**	**gyrB**	**4.48**
**pgi**	**16S rRNA**	**rpoB**	**pgi**	**4.93**	**rpoB**	**rpoB**	**gyrB**	**gyrB**	**5.24**	**gyrB**	**pgi**	**rpoB**	**atpD**	**4.58**
**gyrB**	**infB**	**pgi**	**gyrB**	**6.65**	**gyrB**	**gyrB**	**atpD**	**rpoB**	**5.59**	**infB**	**gyrB**	**atpD**	**pgi**	**6.54**
**infB**	**gyrB**	**gyrB**	**rpoB**	**6.84**	**atpD**	**atpD**	**infB**	**atpD**	**6.26**	**pgi**	**infB**	**infB**	**infB**	**6.65**
**rpoB**	**rpoB**	**infB**	**infB**	**6.95**	**infB**	**infB**	**rpoB**	**infB**	**7.27**	**rpoB**	**rpoB**	**pgi**	**rpoB**	**6.84**
**sodA**	**sodA**	**sodA**	**sodA**	**9.00**	**sodA**	**sodA**	**sodA**	**sodA**	**8.65**	**sodA**	**sodA**	**sodA**	**sodA**	**9.00**

**Table 11:** Candidate reference genes ranked by different methods in serovar C.

Exponential					Stationary					Combined				
geN	Norm	ΔCT	Ranking	Mean	geN	Norm	ΔCT	Ranking	Mean	geN	Norm	ΔCT	Ranking	Mean
**rpoB**	**gyrA**	**rpoB**	**rpoB**	**1.44**	**recN**	**gyrB**	**recN**	**recN**	**1.26**	**gyrA**	**gyrB**	**gyrB**	**gyrB**	**1.44**
**recN**	**recN**	**recN**	**recN**	**2.00**	**gyrB**	**recN**	**gyrB**	**gyrB**	**1.59**	**recN**	**gyrA**	**recN**	**gyrA**	**2.00**
**gyrA**	**rpoB**	**gyrA**	**gyrA**	**2.08**	**rpoB**	**gyrA**	**rpoB**	**rpoB**	**3.56**	**gyrB**	**recN**	**rpoB**	**recN**	**2.29**
**gyrB**	**gyrB**	**gyrB**	**gyrB**	**4.00**	**gyrA**	**pgi**	**gyrA**	**gyrA**	**3.63**	**rpoB**	**rpoB**	**gyrA**	**rpoB**	**3.63**
**16S rRNA**	**16S rRNA**	**16S rRNA**	**16S rRNA**	**5.00**	**pgi**	**rpoB**	**atpD**	**pgi**	**4.93**	**16S rRNA**	**16S rRNA**	**16S rRNA**	**16S rRNA**	**4.64**
**pgi**	**pgi**	**pgi**	**pgi**	**6.00**	**atpD**	**16S rRNA**	**pgi**	**atpD**	**5.94**	**atpD**	**atpD**	**pgi**	**atpD**	**6.00**
**infB**	**infB**	**infB**	**infB**	**7.00**	**16S rRNA**	**atpD**	**16S rRNA**	**16S rRNA**	**6.65**	**pgi**	**pgi**	**atpD**	**pgi**	**6.26**
**atpD**	**atpD**	**atpD**	**atpD**	**8.00**	**infB**	**infB**	**infB**	**infB**	**8.00**	**infB**	**infB**	**infB**	**infB**	**7.65**

### Impact of reference gene selection on gene expression studies

**Fig 2:**
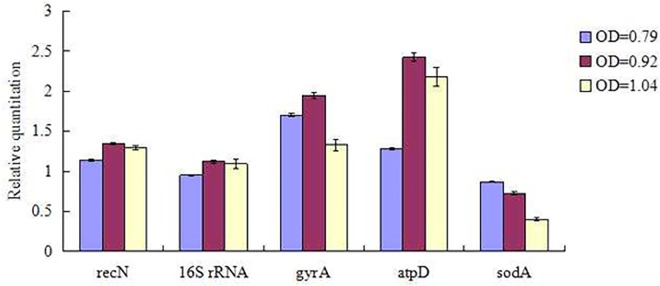
50S ribosomal protein L33 expression analysis at different time points in stationary phase in cultures of serovar B of *A*. *paragallinarum*. Different colored columns show the relative quantification of 50S ribosomal protein L33 when normalized against reference genes at three sampling time points. *RecN* and *16S rRNA* were the two highest ranked genes, *gyrA* and *atpD* were the two highly ranked genes for cultures of serovar B of *A*. *paragallinarum* in stationary phase and *sodA* was a poorly ranked gene.

To determine the effect of a poorly ranked reference gene on a gene expression study, we performed a 50S ribosomal protein L33 expression analysis using data from the cultures of serovar B of *A*. *paragallinarum* in stationary growth phase. Expression of 50S ribosomal protein L33 was assessed using two highly ranked genes: *recN*, 1*6S rRNA*, two middle-ranked reference genes: *gyrA* and *atpD* and a poorly ranked gene-*sodA*. The results revealed a difference in tendency of expression level for 50S ribosomal protein L33 along with the growth time. There was a same tendency when normalised to the two most stable reference genes and two stable reference genes, as opposed to inconsistent result when normalised to the least stable reference gene (Fig 2). Therefore, by using the least stable reference gene *sodA* for normalization significantly changed the calculated expression level of 50S ribosomal protein L33 which could lead to large error alterations in study results.

## Discussion

Real-Time quantitative PCR is among the most powerful tools for detection of expression levels of target genes. Endogenous reference genes are widely used in qPCR assays for normalization because their stability in different experimental conditions and biological treatment system [18,25].Housekeeping genes, such as *18S* and *28S rRNA*, *GAPD (3- GAPDH)*, *ACTB (actin)*, and *TUBLIN (tubulin)*, etc., are often used as reference genes in relative quantification as the proteins encoded s are essential for maintaining cellular activities and are stably expressed in different tissues and organs [25,26]. However, the expression levels of reference genes are only conditionally stable and are subject to different species, experimental conditions, growth phase, biotic and abiotic stimulations. Each particular experimental condition, therefore, has its suitable stable expression reference genes [27–29] and none of the commonly used reference gene is universal [30–32]. For example, four genes, *rpoB*, *atpD*, *gyrA* and *gyrB*, were found to be most stable candidate reference genes; whereas the expression of *16S rRNA*, a commonly used reference gene in many of studies, has been found to be unstable [33]. Furthermore, the expression of some genes varies depending on different growth conditions and stimulations. One study found that *pyk* and *rpoB* performed most stably when comparing aerobic and epinephrine cultures during growth phase; whereas when analyzing exponential and stationary growth phase together, only *pyk* remained in the top three rankings and the *16S rRNA* has been demonstrated unstable under certain study conditions [14]. Choosing a suitable reference gene for gene expression research based on experimental conditions is critical for valid analysis.

In addition, the *atpD* gene we identified is a highly conservative and stable gene in *Pasteurellaceae*, and is commonly used as a molecular biomarker [9] for bacterial categorization and widely used as an reference gene in regenerating phylogenetic tree for *Pasteurellaceae* in recent studies [34]. However, current study is the first to demonstrate its existence in *A*. *paragallinarum*. To provide reference for future studies, we sequenced *A*. *paragallinarum atpD* gene of serovar A, B and C strains and deposited those into GenBank (KXO78457, KXO78458 and KXO78459).

In this study, no universal reference gene was found for all environments and phase. This was probably contributed by variations among different serovars, i.e. the stable reference genes in one serovar might not necessarily be stable in another serovar. Then, the expression level of the same reference gene might vary at different growth phase, such as exponential and stationary phase. Therefore, when choosing reference gene for normalization of expression levels, the timing and serovars need to be taken into consideration for most accurate estimation of expression levels.

The *gyrA* and *rpoB*, which have been evaluated in *Actinobacillus* [14], *Staphylococcus aureus* [35], and *Xanthomona s* [33], and demonstrated most stable expression characteristics, also exhibited stability in our study. *16S rRNA*, a widely used reference gene for species classification, had a low favorably ranking despite its high-abundance with mRNA transcription. Interestingly, compared to other reference genes, *sodA*, which was usually used for strain identification and species classification [10,35], had a high expression level in serovar B, but not in A and C (with CT = 35), and amazingly kept CT values unchanged regardless of assay conditions. This meant that expression levels of *sodA* was very low in serovar A and C beyond the limit of detection for the quantitative PCR, *sodA* was therefore excluded from the ΔCT analysis. In addition, the stabililty ranking of *sodA* was at the bottom for serovar B, which further confirmed that *sodA* was not suitable for normalization in qPCR assays of *A*. *paragallinarum*. The stable ranking of reference genes analyzed by geNorm was generally consistent with that by NormFinder, with minor differences in individual reference genes. For example, the top four stable reference genes analyzed by geNorm were *gyrA*, *atpD*, *rpoB*, and *recN*; while NormFinder recommended the use of *atpD*、*gyrB*、*gyrA*, and *rpoB* for normalization. The variations may be explained by different parameter settings, assumptions and algorithms in geNorm and NormFinder when calculating the gene expression stability of the reference genes. Thus, a combination of two or more software was needed for the stability ranking of candidate genes when selecting reliable reference genes [36].

In conclusion, suitable reference gene candidates were selected for use in serovars A, B, and C of *A*. *paragallinarum* in different growth phase. For serovar A, *gyrA* and *atpD* were the most stably expressed in exponential and stationary phase respectively; for serovar B, *atpD* and *recN* were the most stably expressed in exponential and stationary phase respectively; for serovar C, *rpoB* and *recN* were the most stably expressed in exponential and stationary phase respectively.

## References

[1] Blackall PJ. Infectious Coryza: Overview of the disease and new diagnostic options. Clin Microbiol Rev. 1999; 12(4): 627–632. 10515906 10.1128/cmr.12.4.627 PMC88928

[2] Blackall PJ, Christensen H, Beckenham T, Blackall LL & Bisgaard M. Reclassification of *Pasteurella gallinarum*, *Haemophilus paragallinarum*, *Pasteurella avium* and *Pasteurella volantium* as *Avibacterium gallinarum* gen. nov., comb. nov., *Avibacterium paragallinarum* comb. nov., *Avibacterium avium* comb. nov. and *Avibacterium volantium* comb. nov. Int J Syst Evol Microbiol. 2005; 55(Pt1):353–362. 15653900 10.1099/ijs.0.63357-0

[3] Page LA. *Haemophilus* infections in chickens. 1. Characteristics of 12 *Haemophilus* isolates recovered from diseased chickens. Am J Vet Res. 1962; 23:85–95. 14483162

[4] Blackall PJ, Matsumoto M and Yamamoto R. Infectious coryza In: Calnek BW, Barnes HJ, Beard CW, McDougald LR, Saif YM, editors. Diseases of poultry, 10th ed. Ames: Iowa State University Press; 1997 pp. 179–190.

[5] Huggett J, Dheda K, Bustin S, Zumla A. Real-time RT-PCR normalisation; strategies and considerations. Genes Immun. 2005; 6:279–284. 10.1038/sj.gene.6364190 15815687

[6] Vandecasteele SJ, Peetermans WE, Merckx R, and Van E. Quantification of expression of *Staphylococcus epidermidis* housekeeping genes with Taqman quantitative PCR during in vitro growth and under different conditions. J Bacteriol. 2001; 183:7094–7101. 10.1128/JB.183.24.7094-7101.2001 11717267 PMC95557

[7] Vandesompele J, De Preter K, Pattyn F, Poppe B, Van Roy N, De Paepe A, et al Accurate normalization of real-time quantitative RT-PCR data by geometric averaging of multiple internal control genes. Genome Biol. 2002; 3: research0034.1-research0034.11. 10.1186/gb-2002-3-7-research0034 PMC126239 12184808

[8] Kuhnert P, Korczak BM. Prediction of whole-genome DNA–DNA similarity, determination of G+C content and phylogenetic analysis within the family *Pasteurellaceae* by multilocus sequence analysis (MLSA). Microbiol. 2006; 152 (9): 2537–2548. 10.1099/mic.0.28991-0 16946249

[9] Christensen H, Kuhnert P, Elmerdahl J.O, Bisgaard M, Comparative phylogenies of the housekeeping genes *atpD*, *infB* and rpoB and the *16S rRNA* gene within the *Pasteurellaceae*. International Journal of Systematic and Evolutionary Microbiology. 2004; 54(5), 1601–1609. 15388716 10.1099/ijs.0.03018-0

[10] Christensen H, Bisgaard M. Revised definition of *Actinobacillus sensu stricto* isolated from animals: a review with special emphasis on diagnosis. Vet Microbiol. 2004; 99(1): 13–30. 10.1016/j.vetmic.2003.12.002 15019108

[11] Richardson J, Craighead JC, Cao SL, Handfield M. Concurrence between the gene expression pattern of *Actinobacillus actinomycetemcomitans* in localized aggressive periodontitis and in human epithelial cells. J Med Microbiol. 2005; 54(5): 497–504. 15824431 10.1099/jmm.0.45949-0

[12] Gautier AL, Dubois D, Escande F, Avril JL, Trieu-Cuot P, Gaillot O. Rapid and accurate identification of human isolates of *Pasteurella* and related species by sequencing the *sodA* gene. J Clin Microbiol. 2005; 43(5): 2307–2314. 10.1128/JCM.43.5.2307-2314.2005 15872260 PMC1153776

[13] NØrskov-Lauritsen N, Bruun B, Kilian M. Multilocus sequence phylogenetic study of the genus *Haemophilus* with description of *Haemophilus pittmaniae* sp. nov. Int J Syst Evol Microbiol. 2005; 55(1): 449–456. 15653917 10.1099/ijs.0.63325-0

[14] Bujold AR, MacInnes JI. Validation of reference genes for quantitative real-time PCR (qPCR) analysis of *Actinobacillus suis*. BMC Res Notes. 2015; 8 (1): 86. 25884823 10.1186/s13104-015-1045-8 PMC4369107

[15] Labandeira RM, Mock JR, Hansen EJ. Regulation of expression of the *Haemophilus ducreyi* LspB and LspA2 proteins by CpxR. Infect Immun. 2009; 77: 3402–3411. 10.1128/IAI.00292-09 19451237 PMC2715676

[16] Arvidsson S, Kwasniewski M, Riano-Pachon DM, Mueller-Roeber B. QuantPrime—a flexible tool for reliable high-throughput primer design for quantitative PCR. BMC Bioinformatics. 2008; 9: 465 Available: http://www.biomedcentral.com/1471-2105/9/465. Accessed 2012 Nov 25. 10.1186/1471-2105-9-465 18976492 PMC2612009

[17] Ye J, Coulouris G, Zaretskaya I, Cutcutache I, Rozen S, Madden TL. Primer-BLAST: a tool to design target-specific primers for polymerase chain reaction. BMC Bioinformatics. 2012; 13: 134 Available: http://www.biomedcentral.com/1471-2105/13/134. Accessed 2014 Jan 30. 10.1186/1471-2105-13-134 22708584 PMC3412702

[18] Bustin SA, Benes V, Nolan T, Pfaffl MW. Quantitative real-time RT-PCR—a perspective. J Mol Endocrinol. 2005; 34(3): 597–601. 10.1677/jme.1.01755 15956331

[19] Bustin SA, Benes V, Garson JA, Hellemans J, Huggett J, Kubista M, et al The MIQE guidelines: minimum information for publication of quantitative real-time PCR experiments. Clin Chem. 2009; 55(4): 611–622. 10.1373/clinchem.2008.112797 19246619

[20] Andersen CL, Jensen JL, Orntoft TF. Normalization of real-time quantitative RT-PCR data: a model based variance estimation approach to identify genes suited for normalization-applied to bladder-and colon-cancer data-sets. Cancer Res. 2004; 64: 5245–5250. 10.1158/0008-5472.CAN-04-0496 15289330

[21] Vandesompele J, De Preter K, Pattyn F, Poppe B, Van Roy N, De Paepe A, et al Accurate normalization of real-time quantitative RT-PCR data by geometric averaging of multiple internal control genes. Genome Biol. 2002; 3(7): research0034.1–0034.11. 10.1186/gb-2002-3-7-research0034 PMC126239 12184808

[22] Silver N, Jiang BJ. Selection of housekeeping genes for gene expression studies in human reticulocytes using real-time PCR. BMC Mol Biol. 2006; 7(1):1–9. 17026756 10.1186/1471-2199-7-33 PMC1609175

[23] Chen D, Pan X, Xiao P, Farwell MA, Zhang B. Evaluation and identification of reliable reference genes for pharmacogenomics, toxicogenomics, and small RNA expression analysis. J Cell Physiol. 2011; 226:2469–2477. 10.1002/jcp.22725 21412770

[24] Pfaffl MW, Tichopad A, Prgomet C, Neuvians TP. Determination of stable housekeeping genes, differentially regulated target genes and sample integrity: BestKeeper—Excel-based tool using pair-wise correlations. Biotechnol Lett. 2004; 26(6):509–515. 15127793 10.1023/b:bile.0000019559.84305.47

[25] Gutierrez L, Mauriat M, Guénin S, Pelloux J, Lefebvre JF, Louvet R, et al The lack of a systematic validation of reference genes: a serious pitfall undervalued in reverse transcription-polymerase chain reaction (RT-PCR) analysis in plants. Plant Biotechnol J. 2008; 6(6): 609–618. 10.1111/j.1467-7652.2008.00346.x 18433420

[26] Suzuki T, Higgins PJ, Crawford DR. Control selection for RNA quantitation. Biotechniques. 2000; 29(2): 332–337. 10948434 10.2144/00292rv02

[27] Iskandar HM, Simpson RS, Casu RE, Bonnett GD, Maclean DJ, Manners JM. Comparison of reference genes for quantitative real-time polymerase chain reaction analysis of gene expression in sugarcane. Plant Mol Biol Reporter. 2004; 22(4): 325–337.

[28] Jian B, Liu B, Bi Y, Hou W, Wu C, Han T. Validation of internal control for gene expression study in soybean by quantitative real-time PCR. BMC Mol Biol. 2008; 9(1): 59. 18573215 10.1186/1471-2199-9-59 PMC2443375

[29] Hong SY, Seo PJ, Yang MS, Xiang F, Park CM. Exploring valid reference genes for gene expression studies in *Brachypodium distachyon* by real-time PCR. BMC Plant Biol. 2008; 8(1): 112. 18992143 10.1186/1471-2229-8-112 PMC2588586

[30] Radonić A, Thulke S, Mackay IM, Landt O, Siegert W, Nitsche A. Guideline to reference gene selection for quantitative real-time PCR. Biochem Biophys Res Commun. 2004; 313(4): 856–862. 14706621 10.1016/j.bbrc.2003.11.177

[31] Reid KE, Olsson N, Schlosser J, Peng F, Lund ST. An optimized grapevine RNA isolation procedure and statistical determination of reference genes for real-time RT-PCR during berry development. BMC Plant Biol. 2006; 6(1): 27. 17105665 10.1186/1471-2229-6-27 PMC1654153

[32] Long XY, Wang JR, Ouellet T, Rocheleau H, Wei YM, Pu ZE, et al Genome-wide identification and evaluation of novel internal control genes for Q-PCR based transcript normalization in wheat. Plant Mol Biol. 2010; 74(3): 307–311. 10.1007/s11103-010-9666-8 20658259

[33] Jacob TR, Laia ML, Ferro JA, Ferro MI. Selection and validation of reference genes for gene expression studies by reverse transcription quantitative PCR in *Xanthomonas citri* subsp. citri during infection of *Citrus sinensis*. Biotechnol lett. 2011; 33(6): 1177–1184. 10.1007/s10529-011-0552-5 21318633

[34] Petersen KD, Chistensen H, Bisgaard M, Olsen JE. Genetic diversity of *Pasteurella multocida* fowl cholera isolates as demonstrated by ribotyping and 16S rRNA and partial atpD sequence comparisons. J Microbiol. 2001; 147(Pt 10):2739–2748. 10.1099/00221287-147-10-2739 11577153

[35] Sihto HM, Tasara T, Stephan R, Johler S. Validation of reference genes for normalization of qPCR mRNA expression levels in *Staphylococcus aureus* exposed to osmotic and lactic acid stress conditions encountered during food production and preservation. FEMS Microbiol lett. 2014; 356(1): 134–140. 10.1111/1574-6968.12491 24893820

[36] Jacobsen AV, Yemaneab BT, Jass J, Scherbak N. Reference gene selection for qPCR is dependent on cell type rather than treatment in colonic and vaginal human epithelial cell lines. PLoS One. 2014; 9(12):1–24. 10.1371/journal.pone.0115592 PMC4272277 25526394

